# Genome Sequence of *Fusobacterium nucleatum* Strain W1481, a Possible New Subspecies Isolated from a Periodontal Pocket

**DOI:** 10.1128/genomeA.00009-14

**Published:** 2014-02-13

**Authors:** Mia Yang Ang, David Dymock, Joon Liang Tan, Ming Hang Thong, Qin Kai Tan, Guat Jah Wong, Ian C. Paterson, Siew Woh Choo

**Affiliations:** a Genome Informatics Research Laboratory, University of Malaya, Kuala Lumpur, Malaysia; b Department of Oral Biology and Biomedical Sciences, University of Malaya, Kuala Lumpur, Malaysia; c School of Oral and Dental Sciences, University of Bristol, Bristol, United Kingdom; d Oral Cancer Research and Coordinating Centre, Faculty of Dentistry, University of Malaya, Kuala Lumpur, Malaysia

## Abstract

*Fusobacterium nucleatum* is a bacterial species commonly detected in dental plaque within the human oral cavity, with some strains associated with periodontal disease, one of the most common clinical bacterial infections in the human body. The exact mechanisms of its pathogenesis are still not completely understood. In this study, we present the genome sequence and annotation of *F. nucleatum* strain W1481, isolated from a periodontal pocket of a dental patient at the University of Bristol, United Kingdom, the 16S rRNA gene sequencing of which showed it to be markedly different from the five previously named subspecies.

## GENOME ANNOUNCEMENT

*Fusobacterium nucleatum* is an anaerobic, Gram-negative, and nonsporulating bacterium associated with a wide spectrum of human infections and diseases (1). This species is often found in the oral cavity, as well as in gastrointestinal and genitourinary tracts in humans (2, 3). Recent studies have shown that *F. nucleatum* causes localized infections, such as tonsillitis, peritonsillar abscess, septicemia, and brain, liver, and lung abscesses (4). *F. nucleatum* is more frequently found in the periodontal pocket than in other sites (5), and it has been shown previously that different isolates from different subgroups vary in their pathogenicities and levels of disease activity (6). The species has been subdivided into five subspecies, *F. nucleatum* subsp. *nucleatum, F. nucleatum* subsp. *polymorphum, F. nucleatum* subsp. *animalis, F. nucleatum* subsp. *fusiforme*, and *F. nucleatum* subsp. *vincentii* (7), based on DNA-DNA hybridization comparisons and phenotypic characteristics (8). The representative genome sequences are available for each subspecies (9–16).

In this study, we shotgun sequenced the genome of *F. nucleatum* strain W1481, which was isolated in Cardiff, United Kingdom, in December 1986 from an 8-mm periodontal pocket in a patient with chronic periodontitis, using the Illumina HiSeq 2000 sequencing platform. Based on 16S rRNA and comparative genomic analyses, this strain may represent a new subspecies of *F. nucleatum*. The generated raw genome reads underwent *de novo* assembly using CLC Genomics Workbench version 5.1.5. The assembly of the reads resulted in 186 contigs, with an N_50_ contig size of 44,334 bp. The smallest contig is 418 bp, whereas the largest contig is 135,502 bp. The size of the sequenced genome is 2,477,971 bp, with a G+C composition of 27.6%.

The sequenced genome of *F. nucleatum* W1481 was annotated using the Rapid Annotations using Subsystems Technology (RAST) pipeline (17). RAST predicted 2,163 coding sequences (CDSs) and 56 RNAs in the genome. RAST functional annotation analysis revealed that most of these genes are involved in basic functions, such as cofactor, vitamin, prosthetic group, and pigment synthesis (161 genes), DNA metabolism (56 genes), RNA metabolism (46 genes), and amino acid and derivative synthesis (125 genes). No genes were predicted to be in the functional category of phages, prophages, transposable elements, or plasmids.

Genomic islands (GIs) are clusters of genes in prokaryotic genomes of probable horizontal origin and are often associated with microbial adaptations of medical or environmental interest (18). Using the IslandViewer software (19), we predicted 6 putative GIs in the genome of *F. nucleatum* W1481. An examination of the GIs revealed the presence of genes involved in clustered regularly interspaced short palindromic repeats (CRISPRs) and the restriction-modification (RM) and toxin-antitoxin (TA) systems. According to Makarova et al. (20), all these are defense islands of prokaryotic genomes, which confer resistance to exogenous genetic elements, such as plasmids and phages. PHAST software (21) analysis showed that there are no prophages in the genome of *F. nucleatum* W1481. Therefore, we suggest that the presence of RM and TA systems and CRISPR-associated genes (Cas) in the GIs might confer resistance to the integration of phages into the genome.

In conclusion, we report the genome of *F. nucleatum* W1481. The addition of this new genome may help achieve better insights into the biology, evolution, diversity, and pathogenicity of this oral pathogen.

### Nucleotide sequence accession number.

This whole-genome shotgun project has been deposited at DDBJ/EMBL/GenBank under the accession no. AXUR00000000.
